# Monocytes complexed to platelets differentiate into functionally deficient dendritic cells

**DOI:** 10.1002/JLB.3A0620-460RR

**Published:** 2020-07-14

**Authors:** Meera V. Singh, Sumanun Suwunnakorn, Sydney R. Simpson, Emily A. Weber, Vir B. Singh, Pawel Kalinski, Sanjay B. Maggirwar

**Affiliations:** ^1^ Department of Microbiology and Immunology University of Rochester School of Medicine and Dentistry Rochester New York USA; ^2^ Department of Microbiology and Immunology and Tropical Medicine George Washington School of Medicine and Health Sciences Washington District of Columbia USA; ^3^ Department of Medicine Roswell Park Comprehensive Cancer Center Buffalo New York USA

**Keywords:** CD40L, HIV infection, immunotherapy, platelet‐monocyte complexes

## Abstract

In addition to their role in hemostasis, platelets store numerous immunoregulatory molecules such as CD40L, TGFβ, β2‐microglobulin, and IL‐1β and release them upon activation. Previous studies indicate that activated platelets form transient complexes with monocytes, especially in HIV infected individuals and induce a proinflammatory monocyte phenotype. Because monocytes can act as precursors of dendritic cells (DCs) during infection/inflammation as well as for generation of DC‐based vaccine therapies, we evaluated the impact of activated platelets on monocyte differentiation into DCs. We observed that in vitro cultured DCs derived from platelet‐monocyte complexes (PMCs) exhibit reduced levels of molecules critical to DC function (CD206, dendritic cell‐specific intercellular adhesion molecule‐3‐grabbing nonintegrin, CD80, CD86, CCR7) and reduced antigen uptake capacity. DCs derived from PMCs also showed reduced ability to activate naïve CD4^+^ and CD8^+^ T cells, and secrete IL‐12p70 in response to CD40L stimulation, resulting in decreased ability to promote type‐1 immune responses to HIV antigens. Our results indicate that formation of complexes with activated platelets can suppress the development of functional DCs from such monocytes. Disruption of PMCs in vivo via antiplatelet drugs such as Clopidogrel/Prasugrel or the application of platelet‐free monocytes for DCs generation in vitro, may be used to enhance immunization and augment the immune control of HIV.

## Graphical Abstract

Dendritic cells derived from monocytes complexed to platelets are functionally defective and can negatively impact induction of anti‐HIV immune responses.

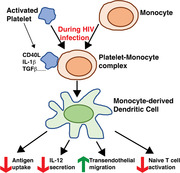

**Abbreviations**
β_2_M: β_2_ microglobulin
ACD: Acid citrate dextrose
DC: Dendritic cells
DC‐SIGN: Dendritic cell‐specific intercellular adhesion molecule‐3‐grabbing nonintegrin
HMDS: Hexamethyldisilazane
MFI: Mean fluorescence intensity
NT: Not treated
Plt: Platelets
PMCs: Platelet‐monocyte complexes
Thr: Thrombin
VSV‐G: Vesicular stomatitis virus G protein

## 1 INTRODUCTION

Dendritic cells (DCs), a heterogeneous population of professional APCs, are crucial inducers of the immune responses against pathogens. In steady state, DC progenitors from bone marrow give rise to circulating and tissue resident DCs, but throughout infections/inflammation, monocytes, which are 20% more abundant than DCs, serve as major DC precursors.^1^ During HIV infection, the virus employs DCs as carriers to disseminate to lymph nodes^2^, ^3^, ^4^ and suppresses DC function and numbers.^5^, ^6^, ^7^, ^8^, ^9^, ^10^, ^11^ We and others have shown that activated platelets form transient complexes with monocytes, termed platelet‐monocyte complexes (PMCs) and the levels of these complexes are increased in the blood of HIV infected individuals.^12^, ^13^, ^14^, ^15^, ^16^ Platelet‐monocyte interaction induces proinflammatory monocytes (characterized by increased expression of CD40, PSGL‐1, and CCR2), capable of migrating across blood brain barrier and cause neuro‐inflammation, thereby exacerbating HIV‐associated neurologic disease.^14^ Activated platelets also mediate the interaction between the endothelium and other leukocytes and can participate in the pathology of various acute and chronic inflammatory conditions.^17^, ^18^, ^19^, ^20^, ^21^ This information raises an important scientific question about the role of platelets in regulating DC‐driven immune responses. In this regard, a few reports have shown that transfusion with platelet concentrates during treatment for thrombocytopenia can modulate DC specific inflammatory response^22^, ^23^, ^24^ and that platelets can regulate DC function via release of biomolecules or direct interaction with DCs.^25^, ^26^, ^27^, ^28^, ^29^

Platelets are small anucleate cells, which store or produce multiple immune mediators including CD40L, TGFβ, β_2_‐microglobulin (β_2_M), platelet factor 4, and IL‐1β, among others. Platelets contain the largest levels of TGFβ in the body and patients with immune thrombocytopenia have low levels of circulating TGFβ.^30^, ^31^, ^32^ In addition, almost 99% of circulating CD40L and most of the β_2_M are of platelet origin.^33^, ^34^, ^35^ These molecules are stored in platelet α‐granules in their bioactive form, and are secreted through the open canalicular system immediately upon cellular activation. Platelets also store highly stable RNAs for molecules such as IL‐1β and contain the complete machinery for splicing and translation.^36^, ^37^, ^38^ Factors such as CD40L, TGFβ, and IL‐1β, are known to modulate DC function. Being in close proximity to platelets in the form of PMCs might significantly increase the exposure of monocytes and their DC progeny to these biomolecules, thus making them prone to platelet‐mediated functional alterations.

In order to test the impact of platelets on DC development, we established an in vitro coculture system in which DCs were derived from platelet complexed vs. noncomplexed monocytes. The resulting immature DCs were matured using two different cytokine cocktails, one which leads to generation of standard DCs^39^ and one which induces their type 1 polarization.^40^, ^41^, ^42^ Our results indicate that in vitro cultured DCs derived from PMCs exhibit reduced levels of several molecules critical to DC function (e.g., CD206, DC‐SIGN, CD80, CD86, CCR7) as well as reduced antigen uptake capacity, deficiency in inducing the proliferation of naïve CD4 and CD8 T cells, and decreased IL‐12 secretion resulting in reduced ability to induce responses to HIV antigens.

## 2 MATERIALS AND METHODS

### 2.1 Ethics statement and demographics

All studies involving human samples were reviewed and approved by the University of Rochester Medical Center Research Subjects Review Board. Blood samples were acquired from adults after written informed consent carried out in accordance of the Declaration of Helsinki. Unless indicated, the donors were healthy individuals, not infected with HIV (*n* = 33). The mean age of donors was 40 (range 18–70). A total of 52.17% participants were males whereas 43.4% were females. In addition, key experiments (data points shown in red color in Figs. 4C, D, and 5) were also performed using blood obtained from HIV infected individuals (*n* = 4, 1 male and 3 females, age range 38–52). Approximately, 40–60 ml of whole blood was collected in acid citrate dextrose (ACD) vacutainers and processed within 2 h of collection. The blood was incubated at room temperature with slow shaking until then.

### 2.2 Reagents and antibodies

Antibodies against CD206 FITC (20 μl, #551135), HLA DR PE (20 μl, #555812), CD80 PE Cy7 (5 μl, #561135), CD40 FITC (20 μl, #555588), CD14 PE (8 μl, #555398), CD16 PE Cy7 (3 μl, 557744), DC‐SIGN FITC (10 μl, #561764), CCR5 PE Cy7 (5 μl, #557752), CCR7 FITC (20 μl, #560548), CD86 PE Cy7 (5 μl, #561128), and CLEC9A PE (1 μl, #563488) were purchased from BD Biosciences (San Jose, CA, USA). Antibodies against CD61 AF647 (5 μl, #336408), MHC I PE (1 μl, #311405), CD163 FITC (15 μl, #333618) and LILRB2 PE (5 μl, #338706) were purchased from Biolegend (San Diego, CA, USA). The titrated amounts per million cells and catalog numbers are mentioned in parentheses. Human recombinant IL‐1β (#102‐LB‐005), IL‐6 (#206‐IL‐010), TNFα (# 210‐TA‐005), IFNγ (#285‐IF‐100), CD40L (#1163‐CL), ELISA kits for IL‐12p70 (called IL‐12 henceforth, #D1200), IL‐10 (#D100B), and HIV‐1 p24 (#DHP240B) were purchased from R&D Systems (Minneapolis, MN, USA). PGE_2_ (#P0409), Poly‐IC (#P1530), recombinant human IL‐2 (#I7908), Polyethyleneimine (#03880), PEG6000 (#81253), thrombin (#T6684‐100UN), and hirudin (#H0393‐100UN) were purchased from Sigma‐Aldrich (St. Louis, MO, USA). Human recombinant IL‐4 (#11846‐HNAE) was bought from Sino Biologicals (Beijing, China) and GM‐CSF (#68‐8777‐82) from Invitrogen (Carlsbad, CA, USA). CD14 microbeads (#130‐050‐201), naïve CD4 T cell isolation kit (#130‐094‐131), and naïve CD8 T cell isolation kit (#130‐093‐244) were purchased from Miltenyi Biotec (San Diego, CA, USA). Human CD3/CD28 Dynabeads (#111.61D), RPMI (#2240071) and Mr. Frosty (#5100‐0001) were purchased from ThermoFisher Scientific (Waltham, MA, USA).

### 2.3 Cell culture

#### 2.3.1 Isolation of platelets

A total of 10 ml of whole blood was centrifuged at 250 ×*g* for 15 min, and platelet‐rich plasma was collected. Following addition of PGI2 (1 μg/ml of platelet‐rich plasma) to maintain platelet quiescence, platelet‐rich plasma was centrifuged at 1000 ×*g* for 10 min to pellet the platelets. The platelet pellet was then washed and resuspended using Tyrode's salt solution (Sigma‐Aldrich) supplemented with ACD anticoagulant and PGI_2_. Subsequently, washed platelets were centrifuged once again at 1000 ×*g* for 10 min, and the remaining purified platelet pellet was resuspended in Tyrode's salt solution without supplements. The purity of isolated platelets was determined using a Sysmex KX‐21N hematology analyzer (Sysmex Inc, Hyogo, Japan) and was found to be 99% pure. The platelets were resuspended at a concentration of 100 million/ml and activated using 1 μg/ml of recombinant human soluble CD40L (henceforth termed as CD40L) at 37°C for 15–20 min.^14^ When indicated, platelets were also activated with thrombin (0.1 U/ml) for 20 min at 37°C. After 20 min thrombin activity was inhibited by adding 0.1 U/ml of hirudin.

#### 2.3.2 Isolation of monocytes

Monocytes were isolated from 30 ml of whole blood. PBMCs were isolated using Ficoll‐Histopaque density gradient centrifugation. Monocytes were isolated using CD14 microbeads (Miltenyi Biotech) as per the manufacturer's instructions.

#### 2.3.3 Isolation of T lymphocytes

Monocyte depleted PBMCs were used to isolate either naïve CD4 T cells or naïve CD8 T cells (using naïve CD4 and CD8 T isolation kits from Miltenyi Biotec). The cells were cryopreserved in RPMI containing 20% FBS and 10% DMSO using Mr. Frosty for overnight at −80°C and were then stored in vapor phase liquid nitrogen freezer until required.

#### 2.3.4 Platelet‐monocyte coculture and differentiation to DCs

Freshly isolated monocytes were cocultured with activated platelets at the ratio of 1:10 and at 1 million monocytes/ml concentration (day 0). Monocytes not mixed with platelets, monocytes treated with CD40L and monocytes treated with Thr/Hir were used as controls. Human recombinant IL‐4 and GM‐CSF (50 ng/ml) were added every alternate day for 5 d to obtain immature DCs. Immature DCs (day 5) were used for flow cytometric analysis of cell surface markers, antigen uptake assay using IgG coated Fluoresbrite microspheres, electron microscopy, and for generating mature DCs. Cells were matured using two different cytokine cocktails for 2 d. Standard cocktail, comprised IL‐1β (25 ng/ml), IL‐6 (2000IU/ml), TNFα (100 ng/ml), and PGE_2_ (2 μg/ml), and is commonly used to generate standard DCs.^39^ Type 1 cocktail comprised IL‐1β (25 ng/ml), TNFα (100 ng/ml), IFNγ (50 ng/ml), IFNα (3000 U/ml), and Poly I:C (20 μg/ml), which is known to drive DCs toward a type 1 phenotype.^40^, ^41^, ^42^ Immature and mature DCs were washed three time with RPMI containing 10% FBS prior to performing all the assays, in order to remove the growth factors, unbound platelets and HIV.

### 2.4 Flow cytometry

Immature and mature DCs were processed for flow cytometric analysis on days 5 and 7, respectively. The cells were washed once with staining buffer (PBS with 0.1% BSA) and stained with titrated amounts of antibodies against CD61, CD14, CD16, CD206, HLADR, CD80, CD40, DC‐SIGN, MHCI, CCR5, CCR7, CD86, LILRB2, CCR7, CLEC9A, and CD163. The cells were acquired using BD Accuri C6 flow cytometer BD Biosciences, San Jose, CA, USA. A total of 10,000 gates events were acquired on forward and side scatter plot at slow rate. The results were analyzed using Flow Jo software (version 10). For analysis, cells were first gated on forward and side scatter, followed by expression of CD61, a platelet marker (graphs from one representative experiment are shown in Supporting Information Fig. S1A). Data is shown as fold change of percentage of cells expressing respective markers and/or mean fluorescence intensity (MFI) as compared to nontreated (NT) cells.

### 2.5 Antigen uptake assay

Immature DCs (day 5) were incubated with IgG‐coated fluorescent beads (1 μm Fluoresbrite YG carboxylate microspheres) for 45 min at 37°C to allow for phagocytosis.^43^  The phagocytosis was stopped by adding ice cold PBS. Cells were placed on ice and stained with antibodies against CD61. Internalized beads were distinguished from externally bound beads by using PE‐conjugated anti‐human IgG. Cells were then washed and analyzed by flow cytometry as described earlier.

### 2.6 Electron microscopy

#### 2.6.1 Scanning electron microscopy (SEM)

Immature DCs complexed to platelets (day 5) were allowed to adhere onto poly‐L‐lysine–coated coverslips for 2 h and were subsequently placed into 0.1 M sodium cacodylate–buffered 2.5% glutaraldehyde at 4°C for overnight fixation. The cells on the cover glasses were post‐fixed using the same buffer in 1.0% osmium tetroxide and then transitioned through a graded series of ethanol to 100% (×2), then through a graded series of 100% ethanol/hexamethyldisilazane (HMDS) and finally into three changes of pure HMDS. The last change was allowed to evaporate off of the cover glasses overnight in a fume hood. The cover glasses were then mounted onto aluminum stubs and sputter coated with gold. Imaging was performed using a Zeiss Auriga field emission SEM (Carl Zeiss AG, Oberkochen, Germany).

#### 2.6.2 Transmission electron microscopy (TEM)

Immature DCs complexed to platelets (day 5) were allowed to adhere to a two‐chamber slide for 2 h, following which media was removed and immediately replaced with room temperature fixative composed of 0.1 M sodium cacodylate–buffered 2.5% glutaraldehyde. The slides were fixed for 1 h at room temperature and then at 4°C overnight. The plastic chambers were removed, and slides were rinsed in the same buffer, post‐fixed in 1.0% osmium tetroxide for 30 min, and then dehydrated through a graded series of ethanol to 100% concentration. The slides were then placed into a 1:1 ratio of 100% ethanol and Spurr epoxy resin for 1 h and transferred to Spurr epoxy resin overnight. The next day, size 3 BEEM capsules were filled with Spurr resin, inverted, and placed on top of the area where cells were present on the slides, and then placed into a 60°C oven to allow for overnight polymerization. The following day, glass slides/capsules were dipped three to four times in liquid nitrogen, and polymerized BEEM capsules were wiggled and “popped off” the glass. The epoxy blocks with the entrapped cells were trimmed of excess plastic resin, placed into an ultramicrotome, thin sectioned at 70 nm onto carbon‐coated nickel grids, and stained with uranyl acetate and lead citrate. Imaging was done using a Hitachi 7650 transmission electron microscope with an attached Gatan Erlangshen 11‐megapixel digital camera (Gatan Inc, Pleasanton, CA, USA).

### 2.7 3D flow chamber migration assay

The assay was performed as described previously.^44^ Briefly, 5 μm pore membranes (Expedeon Ltd, Abcam, Cambridge, UK) were treated with 50 μg/mL of collagen for 1 h at room temperature, then seeded with 4 × 10^4^ HUVEC cells/insert and allowed to form a monolayer for 24 h at 37°C. A peristaltic pump and 3D flow chamber were obtained from Ismatec (Wertheim, Germany) and C.B.S. Scientific Company, Inc. (San Diego, CA, USA), respectively. The chamber consists of an upper compartment and lower compartment, separated by a membrane prepared as described earlier. Media with 100 ng/mL CCL19 was added to each lower chamber to create a chemokine gradient prior to the addition of the membrane. DCs were cultured in presence or absence of CD40L treated platelets and matured with two different cocktails as described earlier. Cells derived from PMCs were labelled with CellTrace CFSE (ThermoFisher Scientific) and cells derived from noncomplexed monocytes were labelled with CellMask Red (ThermoFisher Scientific) according to the manufacturer's protocol. Labelled noncomplexed and platelet complexed DCs for each individual's cocktail were combined together (2.5 × 10^5^ cells/group) in a total volume of 20 ml. Cells were run through the 3D flow chamber device for 60 min using a flow rate of 0.9 mL/min (2.9 dyne/cm^2^). The 3D flow chamber was disassembled. Transmigrated cells were harvested from the lower compartment of the wells, fixed in 4% PFA, and analyzed by volumetric flow cytometry using Accuri C6 flow cytometer (BD Biosciences).

### 2.8 IL‐12 and IL‐10 ELISA

DCs were matured using the standard and type 1 cocktail as described earlier. On day 7, culture supernatant was harvested (called pre‐CD40L) and was used to measure IL10 and IL12 levels by ELISA as per manufacturer's instructions (Quantikine ELISA kits, R&D Systems). On the same day, after harvesting the pre‐CD40L supernatants, cells were washed three times with RPMI containing 10% FBS to remove the growth factors and were stimulated with CD40L (1 μg/ml) for 24 h. The culture supernatant which was harvested after 24 h of CD40L treatment (called as post‐CD40L) was also used for IL‐12 and IL‐10 ELISAs.

### 2.9 Generation of HIV‐1 virus stock

HIV‐1 NL4‐3 ΔEnv reporter vector was obtained through the NIH AIDS Reagent Program (# 11100).^45^ Plasmid expressing vesicular stomatitis G protein (VSV‐G) was obtained from Dr. Dykes.^46^ The virus stocks were generated as described previously with some modifications.^47^, ^48^ Briefly, human embryonic kidney cells, 293T, were cotransfected with 40 μg each of NL4‐3 vector and VSV‐G vector using polyethyleneimine. Virus supernatant was harvested after 72 h and concentrated using PEG6000 as described and resuspended in PBS. HIV‐1 p24 protein quantitation was performed by ELISA according to manufacturer's instructions.

### 2.10 T cell stimulation

Autologous naïve CD4 or CD8 T cells were revived a day before setting up the assay and were incubated at 37°C overnight in RPMI containing 10%FBS and 20 U/ml of IL‐2. Next day, the T cells were labelled with CFSE as per the manufacturer's instructions. DCs matured using each cytokine cocktail were pulsed with VSV‐G pseudotyped, single cycle replication, HIV isolate for 2 days (500 ng/ml of p24 per million cells). Cells were washed three times with RPMI containing 10% FBS to remove the growth factors and mixed with the T cells in the ratio of 1:10 and at the concentrations of 0.25 million cells/ml. The mixed cultures were incubated at 37°C for 7 d. Half of the medium was replaced with fresh medium containing 20 U/ml IL‐2 on day 3. On day 7, the cells were harvested and analyzed by volumetric flow cytometry to measure the number of CFSE^lo^ cells. T cells not labelled with CFSE and CFSE labelled T cells, stimulated with CD3/CD28 beads were used as controls. Supernatants from day 3 and day 7 post‐DC‐CD4 T cell cocultures were used to measure p24 levels by ELISA.

### 2.11 Statistical analysis

GraphPad Prism v7 was used for statistical analysis. The data are shown as scatter data plots with the error bar representing mean and sd. Normality analysis was done by Shapiro‐Wilk normality test. All the data passed the normality test. It was further analyzed by repeated measures 1‐way ANOVA or 1‐way ANOVA followed by Tukey's multiple comparisons test. Paired *t*‐test was used analyze migration assay. The symbol ^*^indicates *P* < 0.05, ^**^indicates *P* < 0.01, ^***^indicates *P* < 0.001, and ^****^indicates *P* < 0.0001.

## 3 RESULTS

### 3.1 Immature DCs derived from PMCs exhibit reduced antigen uptake ability

**FIGURE 1:**
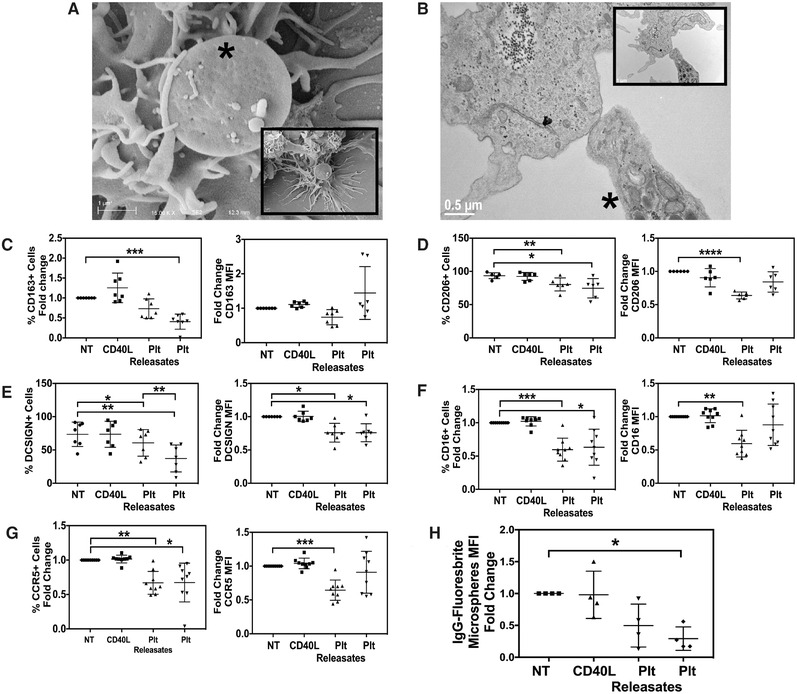
**Phenotypic characterization and evaluation of antigen uptake by immature dendritic cells (DCs)**. Monocytes cultured in presence or absence of activated platelets were differentiated into immature DCs using IL4 and GM‐CSF (50 ng/ml each) for 5 d. (**1A** and **1B**) Scanning electron microscopy and transmission electron microscopy images of platelet‐DC complexes. The platelet is marked by a black star symbol. (**1C**–**1H**) DCs that were derived from monocytes cocultured with platelets and expressed CD61, a platelet marker, are termed “Plt.” DCs that were derived from monocytes cocultured with platelets, did not express CD61, but were still exposed to platelet‐derived biomolecules are called “Plt Releasates.” DCs derived from noncomplexed monocytes (not treated; NT), and CD40L treated monocytes (CD40L) were used as controls. Cells from each group were stained with respective monoclonal antibodies and analyzed using Accuri C6 flow cytometer and FlowJo software v10. The data is shown as raw data or fold change as compared to NT (HIV uninfected individuals, *n* = 6–9, 4 males and 5 females). Results were analyzed by repeated measures 1‐way ANOVA followed by Tukey's multiple comparisons test using GraphPad prism v7. ^*^ indicates *P* < 0.05, ^**^indicates *P* < 0.01, and ^***^indicates *P* < 0.00. Percentages and mean fluorescence intensity (MFI) for expression of (**1C**) CD163, (**1D**) CD206, (**1E**) DC‐SIGN, (**1F**) CD16, and (**1G**) CCR5 on DCs using Accuri C6 flow cytometer. (**1H**) MFI for phagocytosed IgG‐Fluoresbrite microspheres by immature DCs

DCs employ multiple receptors to capture antigens^43^ and in the first set of experiments (HIV uninfected individuals, *n* = 6–9 donors, 4 males, 5 females), we measured the expression of these receptors on immature DCs derived from PMCs as well as noncomplexed monocytes. Briefly, monocytes were cultured with autologous platelets (activated using CD40L) at the ratio of 1:10 for 5 d in presence of IL4 and GM‐CSF. Monocytes cultured alone (NT) and monocytes treated with CD40L were used as treatment controls (CD40L). On day 5, flow cytometric analysis indicated that among the cells derived from platelet‐monocyte cocultures, approximately 30–40% DCs were complexed to platelets (Plt) as seen by expression of CD61, a platelet marker, and the rest of the cells, although not complexed to a platelet physically, were still exposed to platelet‐derived biomolecules (CD61^−^ DCs, Plt releasates). The other two treatments groups had 3% or less of platelet‐DC complexes (Supporting Information Fig. S1B). SEM and TEM analysis of platelet‐DC complexes revealed that platelet and DC cell membranes were in very close proximity to each other indicating a possible interaction between these two cell types via cell surface proteins (Fig. 1A and B, respectively, the platelet is marked by ^*^).

As compared to NT DCs, platelet complexed DCs had significantly reduced percentage of cells expressing CD163 (Fig. 1C, *P* = 0.0007), CD206 (Fig. 1D, *P* = 0.0233), DC‐SIGN (Fig. 1E, *P* = 0.0016), and CD16 (Fig. 1F, *P* = 0.0151). DC‐SIGN also showed significantly reduced MFI of expression in platelet complexed DCs (*P* = 0.0129). Interestingly, DCs that were exposed to platelet releasates showed significantly reduced MFIs for CD206 (*P* < 0.0001), DC‐SIGN (*P* = 0.0161), and CD16 (*P* = 0.0014). Further these cells also showed reduced percentages of cells expressing CD206 (*P* = 0.0063), DC‐SIGN (*P* = 0.0123), and CD16^+^ cells (*P* = 0.0005), indicating that platelet‐origin biomolecules can alter DC phenotype even without direct physical contact. Further, platelet complexed DCs and DCs exposed to platelet biomolecules, showed reduced expression of CCR5, a coreceptor for HIV, indicating that these cells might be less efficient at interacting with this pathogen (Fig. 1G, *P* = 0.0342 and 0.0015 respectively). There was no difference between CD40L treated DCs and the other treatment groups. Raw data for markers depicted in fold change is shown in Supporting Information Figure S1C–G. Further, we measured the expression of other DC markers such as MHCI, HLADR, CD40, CD80, CD86, CCR7, CLEC9A, and LILRB2 on the immature DCs. These markers are functionally important in mature DCs as they play a role in antigen presentation, costimulation, migration to lymph node, among others. Results showed that platelet DC complexes showed reduced percentages of HLA DR^+^ and CD80^+^ cells as well as reduced MFI for CD80 (Supporting Information Fig. S1H, I).

Last, in order to assess if reduced expression of the antigen uptake receptors affects the phagocytotic ability of these DCs, an antigen uptake assay was performed using Fluoresbrite YG Carboxylate microspheres (HIV uninfected individuals, *n* = 4 donors, 2 males, 2 females). Platelet complexed DCs showed significantly reduced internalization of these beads as compared to NT DCs (Fig. 1H, *P* = 0.0139) and there was no difference between CD40L treated DCs and the other treatment groups. These findings indicate that formation of complexes with platelets can negatively impact the differentiation of monocytes into immature DCs.

### 3.2 Platelet complexed DCs mature into phenotypically inferior DCs as compared to noncomplexed DCs

Antigenic stimuli trigger the process of DC maturation, which is associated with increased expression of MHC molecules and costimulatory molecules followed by migration to lymph nodes and terminal differentiation to initiate a T cell immune response. Various combinations of cytokines have been used in vitro to mimic this complex process in monocyte derived DCs (MoDCs). The most commonly used cocktail, comprises IL‐1β, IL‐6, TNFα, and PGE_2_ (standard cocktail).^39^ In this experiment, the immature DCs were matured for an additional 48 h (day 7) using this cocktail and followed by phenotypic characterization (HIV uninfected individuals, *n* = 6‐9 donors, 6 males and 3 females).

**FIGURE 2:**
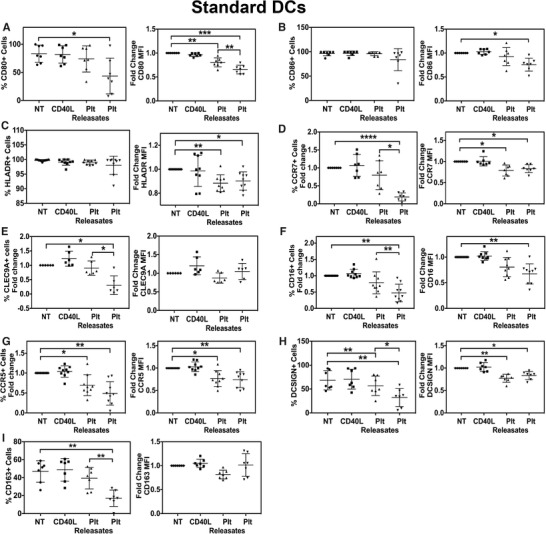
**Phenotypic characterization of dendritic cells (DCs) matured using standard cocktail**. (**2A**–**2I**) Immature DCs were obtained as described in methods section. Cells were matured using standard cocktail comprising IL‐1β (25 ng/ml), IL‐6 (2000 IU/ml), TNFα (100 ng/ml), and PGE_2_ (2 μg/ml) for two more days followed by flow cytometric analysis for expression of various cell surface proteins (HIV uninfected individuals, *n* = 6–9, 6 males and 3 females). Results were analyzed by repeated measures 1‐way ANOVA followed by Tukey's multiple comparisons test using GraphPad prism v7. ^*^indicates *P* < 0.05, ^**^indicates *P* < 0.01, ^***^indicates *P* < 0.001, and ^****^indicates *P* < 0.0001. Percentages and mean fluorescence intensity (MFI) for expression of (**2A**) CD80, (**2B**) CD86, (**2C**) HLA DR, (**2D**) CCR7, (**2E**) CLEC9A, (**2F**) CD16, (**2G**) CCR5, (**2H**) DC‐SIGN, and (**2I**) CD163

Results indicate that similar to immature DCs, about 40% of the mature DCs were found complexed to platelets on day 7 (Supporting Information Fig. S2A), whereas NT and CD40L treated DCs showed less than 2% complexes. DCs complexed to platelets exhibited reduced percentage of CD80^+^ (Fig. 2A, *P* = 0.0133), CCR7^+^ (Fig. 2D, *P* < 0.0001), and CLEC9A^+^ (Fig. 2E, *P* = 0.0128) cells as compared to noncomplexed (NT) cells. These cells also showed reduced MFI for CD80 (*P* = 0.0002), CD86 (Fig. 2B, *P* = 0.018), HLA DR (Fig. 2C, *P* = 0.0211), and CCR7 (*P* = 0.0121). Further, DCs that were not complexed to platelets but were exposed to their releasates, showed reduced MFI for CD80 (*P* = 0.0063), HLA DR (*P* = 0.0046), and CCR7 (*P* = 0.0189), reiterating the fact that physical contact with platelets might not be strictly necessary for induction of platelet mediated changes in DC surface protein expression; however, the effect is more pronounced if they are attached to platelets. Raw data for these markers is shown in Supporting Information Figure S2B–F.

Next, we measured the expression different antigen uptake receptors on mature DCs because a report by Platt et al. showed that contrary to previous beliefs, mature DCs continue to capture, process, and present antigens via endocytotic receptors.^43^ Platelet complexed DCs showed reduced percentage and MFI for CD16 (Fig. 2F, *P* = 0.0017 and *P* = 0.0046), CCR5 (Fig. 2G, *P* < 0.0035 and *P* = 0.0092), DC‐SIGN (Fig. 2H, *P* = 0.0039 and *P* = 0.0105), and percentage for CD163 (Fig. 2I, *P* = 0.003), indicating that these cells have a lower antigen uptake ability as compared to noncomplexed DCs. Raw data for these markers is shown in Supporting Information Figure S2G–I.

**FIGURE 3:**
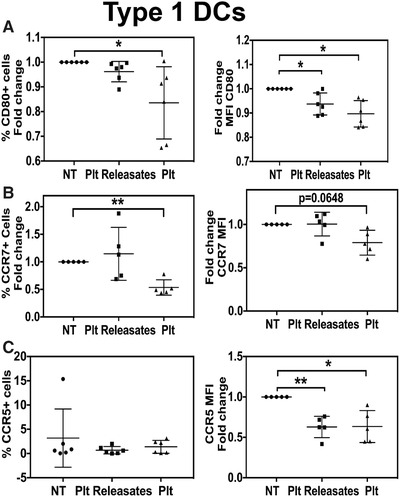
**Phenotype of dendritic cells (DCs) matured using type 1 cocktail**. (**3A**–**3C**) Immature DCs were obtained as described in methods section. Cells were matured using type 1 cocktail comprising IL‐1β (25 ng/ml), TNFα (100 ng/ml), IFNγ (50 ng/ml), IFNα (3000 U/ml), and Poly I:C (20 μg/ml) for two more days followed by flow cytometric analysis for expression of various cell surface proteins (HIV, uninfected individuals, *n* = 5–6, 2 males and 4 females). Results were analyzed by repeated measures 1‐way ANOVA followed by Tukey's multiple comparisons test using GraphPad prism v7. ^*^indicates *P* < 0.05 and ^**^indicates *P* < 0.01. Percentages and mean fluorescence intensity (MFI) for expression of (**3A**) CD80, (**3B**) CCR7, and (**3C**) CCR5

Recently, there have been speculations about the functional quality of DCs generated using the standard cocktail. For example, these DCs fail to secrete IL‐12p70 upon stimulation with CD40L, which is a very important attribute that defines their ability to induce CTL immune responses.^40^ We have previously shown that a cytokine cocktail consisting of IL‐1β, TNFα, IFNγ, IFNα, and Poly I:C can yield stable, type‐1 polarized DC that produce up to 100‐fold higher levels of IL‐12p70 and can induce very high levels of melanoma‐specific CTLs as compared to standard DCs.^40^ Hence in the next set of experiments, we assessed expression of the earlier‐mentioned cell surface molecules in platelet complexed DCs that were matured using the type‐1 cocktail (HIV uninfected individuals, *n* = 5‐6 donors, 2 males and 4 females). On day 7, flow cytometric analysis showed that despite the use of such a potent cytokine cocktail, platelet complexed DCs exhibited reduced percentages and MFIs for CD80 (Fig. 3A, *P* = 0.045 and *P* = 0.0129) and reduced percentage for CCR7 (Fig. 3B, *P* = 0.0038) and a reduced MFI for CCR5 (Fig. 3C, *P* = 0.0313). Raw data for these markers is shown in Supporting Information Figure S3A–C. We did not see any statistically significant difference in the expression of other markers among the different treatment groups in type 1 DCs (CD86, HLA DR, CLEC9A, CD16, DC‐SIGN, and CD163). These results demonstrate that maturation of platelet complexed DCs using two different cytokine cocktails generated phenotypically aberrant DCs, with standard PMC‐DCs showing a more prominent defect.

Please also note that for all experiments henceforth, the “Plt” cells are a mixed population of platelet complexed DCs (approximately 40%) and cells exposed to platelet origin biomolecules (because they were cocultured in the same environment).

### 3.3 Complex formation with platelets increases the ability of DCs to migrate across endothelial barrier

**FIGURE 4:**
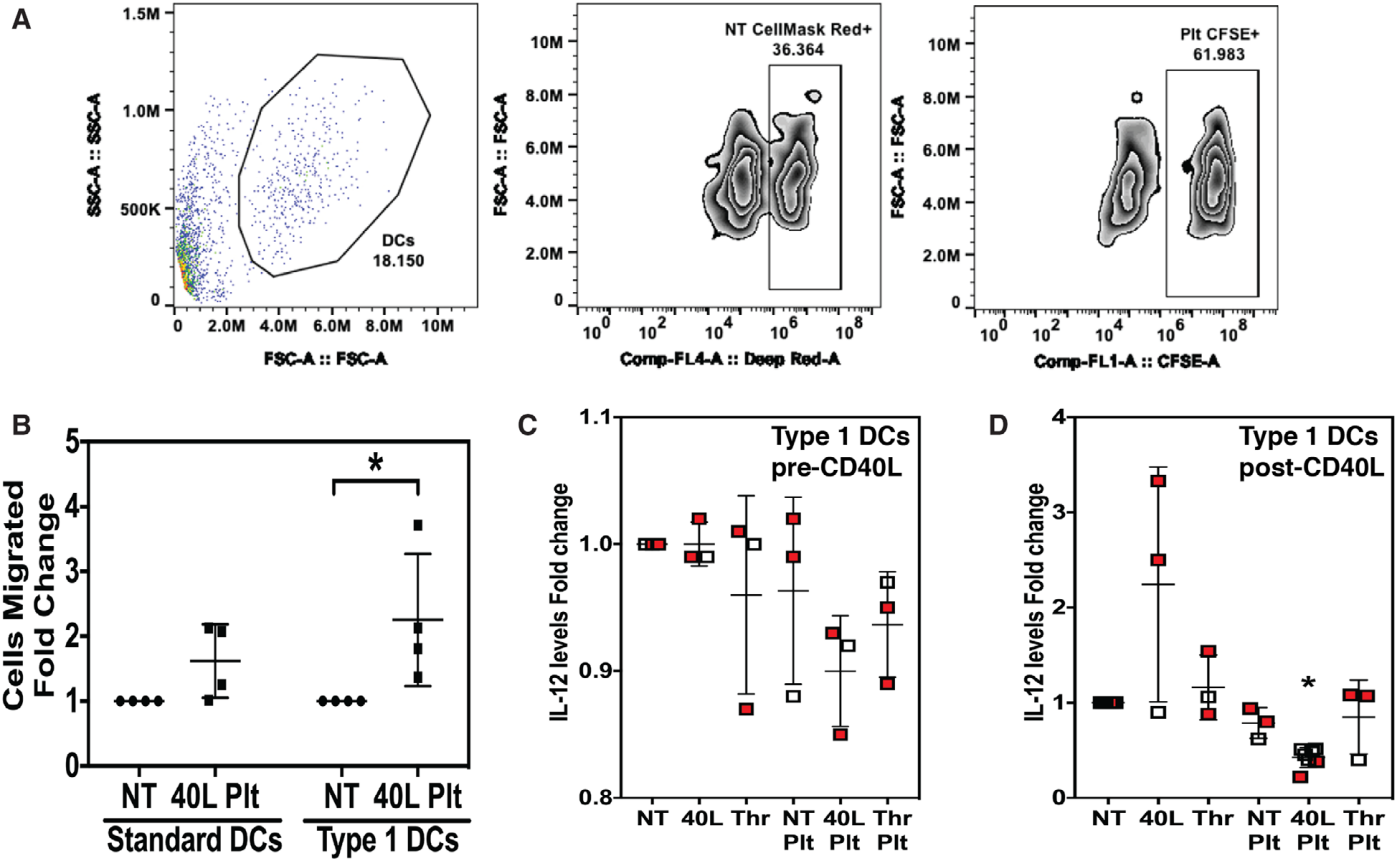
**Evaluation of dendritic cell (DC) functionality: migration across endothelium and cytokine production**. (**4A**–**4B**) DCs matured using both the cocktails were subjected to migration toward CCL19 gradient across a monolayer of endothelial cells using 3D flow chamber device (*n* = 3–4, 2 males and 2 females). Not treated (NT) DC were labelled with CellMask Red and Plt DC with CFSE. Equal numbers of NT and Plt cells were mixed and were flowed through the device for 60 min using a flow rate of 0.9 mL/min (2.9 dyne/cm^2^). The cells that had migrated through to the lower chamber were collected and counted using volumetric analysis on Accuri C6 flow cytometer. (**4A**) Flow plots from one representative experiment (**4B**) Fold change in the number of migrated cells as compared to NT. (**4C**–**4D**) Culture supernatant from mature type 1 DCs before and after CD40L (1 μg/ml for 24 h) treatment was used to measure IL‐12 levels by ELISA (HIV infected and uninfected individuals, *n* = 3–7, 3 males and 4 females). Fold change for the levels of IL‐12 (**4C**) pre‐CD40L and (**4D**) post‐CD40L treatment as compared to NT. Data obtained from HIV infected subjects is denoted in red. Results were analyzed by paired *t*‐test or 1‐way ANOVA followed by Tukey's multiple comparisons test using GraphPad prism v7. ^*^ indicates *P* < 0.05

Upon encountering an antigen, DCs migrate to the lymph node and present the antigen to lymphocytes. CCR7 acts as the receptor for CCL19, which is the most predominant chemokine that is involved in the migration of DCs to lymph node. Our flow cytometry results showed that platelet complexed DCs expressed reduced levels of CCR7 upon maturation with the two cytokine cocktails (Figs. 2D and 3B), indicating they might be less efficient in migrating toward CCL19. On the contrary, activated platelets are very proficient at adhering to endothelial cells, creating a possibility that they might override the CCR7‐CCL19 signal. Hence in the next experiment we used a 3D flow chamber device to measure the migration of DCs across endothelial cell layer toward CCL19 chemokine gradient (HIV uninfected individuals, *n* = 3‐4, 2 males and 2 females). DCs derived from noncomplexed monocytes (NT) and DCs derived from monocytes cocultured with CD40L treated platelets (40L Plt) were used for this experiment. Migrated cells were counted using Accuri C6 flow cytometer. Figure 4A shows flow cytometry data plots for migrated cells from one representative sample. The results indicate that standard DCs derived from platelet cocultured monocytes show a trend toward increased migration in two out of four donors, whereas for type 1 DCs, there was a significant increase in the numbers of migrating, platelet exposed DCs (Fig. 4B, *P* = 0.0498). These results indicate that despite the loss in CCR7 expression, platelet bound DCs can migrate toward the CCL19 chemokine gradient with higher efficiency than noncomplexed DCs. Raw data for this experiment is shown in Supporting Information Figure S3D.

### 3.4 DCs derived from monocytes complexed to activated platelets exhibit decreased ability to secrete IL‐12

**FIGURE 5:**
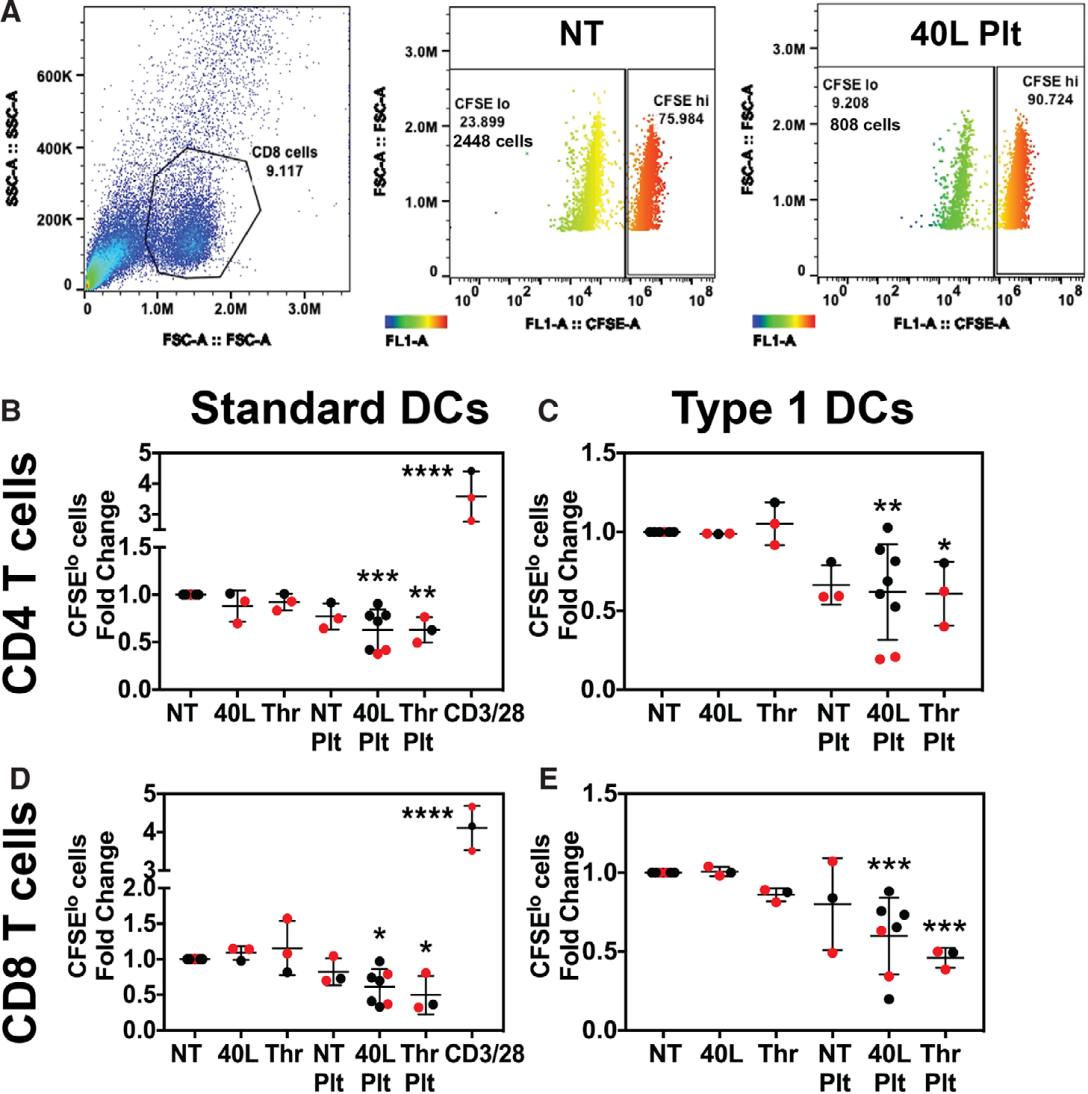
**T cell activation by mature dendritic cells (DCs) derived from platelet complexed and noncomplexed monocytes**. Immature DCs obtained from monocytes that were either complexed with not‐activated, CD40L activated or thrombin activated platelets or reagent controls were pulsed with vesicular stomatitis virus (VSV) pseudo‐typed, single cycle replication recombinant HIV and exposed to the two maturation cocktails for 2 d. Naïve, autologous CD4 or CD8 T cells were labelled with CFSE and mixed at the ratio of 10:1 with DCs. The mixed culture was incubated for seven more days followed by measurement of CFSE^lo^, that is, proliferated T cells using volumetric flow analysis (HIV infected and uninfected individuals, *n* = 3–8, 4 males and 4 females). Data obtained from HIV infected subjects is denoted in red. Results were analyzed by 1‐way ANOVA followed by Tukey's multiple comparisons test using GraphPad prism v7. ^*^indicates *P* < 0.05, ^**^ indicates *P* < 0.01, ^***^indicates *P* < 0.001, and ^****^indicates *P* < 0.0001. (**5A**) Flow plots from one representative experiment for CD8 T cells. Fold change for CFSE^lo^ CD4 T cells as compared to not treated (NT) for (**5B**) standard and (**5C**) type 1 DCs. Fold change for CFSE^lo^ CD8 T cells as compared to NT for (**5D**) standard and (**5E**) type 1 DCs

In the next set of experiments we measured different readouts of DC function such as cytokine secretion and ability to stimulate naïve, autologous CD4 and CD8 T cells. In order to further confirm that platelet activation was indeed an important aspect of platelet‐mediated regulation of DC function, we used DCs differentiated in presence of not‐activated platelets (NT‐Plt), Platelets activated using CD40L (40L‐Plt) and platelets activated using thrombin (Thr Plt). In addition, we also used cells derived from two HIV infected individuals (denoted as red colored data points in respective graphs of Figs. 4 and 5 and Supporting Information Fig. S3). Inclusion of these two participants was done to investigate if MoDCs obtained from HIV infected individuals also showed similar defects when derived from PMCs.

Cell culture supernatants obtained from these cells were used to measure the levels of IL‐12 and IL‐10 secreted by DCs (*n* = 3‐7 donors, of these 2 were infected with HIV, 3 males and 4 females). The ability of DCs to secrete IL‐12 upon receiving CD40 signals is an important clinical readout of their function because IL‐12 producing cells are better able to stimulate CTLs.^49^ We also measured IL‐10 produced by these cells because this cytokine is associated with induction of regulatory T cells or anergic T cells. Cell culture supernatant that the cells had been cultured in for 7 d of DC differentiation/maturation process was used to measure IL‐10 and IL‐12 levels (termed as pre‐CD40L). In addition, DCs were stimulated with CD40L and the supernatant obtained post stimulation (post‐CD40L) was also used to measure IL‐12 and IL‐10. As expected,^40^ standard DCs did not secrete detectable levels of IL‐12 in either pre‐CD40L or post‐CD40L conditions (data not shown). Type 1 DCs cocultured with CD40L and thrombin activated platelets showed a trend toward reduced levels of IL‐12 (not statistically significant, Fig. 4C). When stimulated with CD40L, similar results were obtained with only 40LPlt‐DCs reaching statistical significance (Fig. 4D and Supporting Information Fig. S3E, *P* = 0.0121). There was no statistically significant difference in the levels of IL‐10 in either cocktail or treatment groups, pre‐ or post‐CD40L stimulation. However, DCs cultured in presence of activated platelets tended toward secreting higher levels of IL‐10 (Supporting Information Fig. S3F, G). These results indicate that upon contact with activated platelets, there is a reduction in the ability of DCs to secrete IL‐12, both in HIV infected and uninfected individuals.

### 3.5 PMC‐DCs exhibit reduced capacity to induce HIV‐specific responses

Finally, we measured the ability of DCs to activate naïve CD4 and CD8 T cells (*n* = 3‐8, of these 2 were infected with HIV, 4 males and 4 females). For this purpose, immature DCs were pulsed with VSV‐G pseudo‐typed, single cycle replication, recombinant HIV and exposed to each of the cytokine cocktails. These cells were used in in vitro sensitization (IVS) with autologous, CFSE‐labeled, naïve CD4 or CD8 T lymphocytes. Flow cytometry plots from one representative experiment are shown in Figure 5A. DCs derived from monocytes that were cocultured with CD40L or thrombin activated platelets, showed a significantly reduced capacity to stimulate the proliferation of naïve CD4 T cells as compared to DCs derived from noncomplexed monocytes for standard DCs (Fig. 5B, *P* = 0.0005 40L Plt and 0.0062 Thr Plt) as well as type 1 DCs (Fig. 5C, *P* = 0.0025 for 40L Plt and 0.0241 for Thr Plt). The only exception to this result was data obtained from one HIV‐ donor specifically with respect to DCs cultured with 40L‐Plt. Similar results were obtained when CD8 T cells were stimulated using standard DCs (Fig. 5D, *P* = 0.0116 for 40L Plt and 0.0106 for Thr Plt) and type 1 DCs (Fig. 5E, *P* = 0.0008 for 40L Plt and 0.0005 for Thr Plt). The raw data is shown in Supporting Information Figure S3H and I. DCs obtained using monocytes that were cocultured with not activated platelets also showed defects in T cell stimulation to some extent but it did not reach statistical significance. Cells obtained from HIV infected donors also showed a similar loss in T cell stimulation by DCs derived from PMCs. We also measured p24 levels in day 3 and day 7 post‐DC‐CD4 T cell coculture supernatants, in order to make sure that the VSV pseudo‐typed virus that was used as a source of antigen for the DCs, did not infect CD4 T cells resulting in reduced number of proliferating T cells. The levels of p24 detected were very negligible (Supporting Information Fig. S3J) and we did not see any increase in p24 levels from day 3 to day 7.

These results indicate that platelets, particularly, activated platelets impair the differentiation and maturation of monocytes (that they are complexed with) into functional DCs, enhance their migration across the endothelium and that these cells are less efficient at stimulating the proliferation of naïve CD4 and CD8 T cells in response to HIV antigen. This phenomenon can be observed using cells isolated from both HIV infected and uninfected donors.

## 4 DISCUSSION

More than 35 yr after the identification of HIV and despite massive research efforts, RV144 is the only vaccine so far that has showed 31% reduction in new infections.^50^ However, its efficacy is transient and insufficient to allow the use of this vaccine outside of clinical trial setting. Administration of ex vivo generated DCs is one of the lucrative strategies to overcome the natural deficit in immunity against the virus^51^, ^52^, ^53^, ^54^, ^55^, ^56^, ^57^, ^58^, ^59^, ^60^ and to drive the immunity in desired direction, that is, type 1 immunity, which can potentially control HIV.^50^, ^61^, ^62^ The rationale for active immunization is further supported by the observations that spontaneous, HIV‐specific immune responses are exhibited by a select group of individuals who are able to control virus replication without any antiretroviral treatment (HIV controllers or nonprogressors). Studies have suggested that DCs from controllers are better able to capture antigens as well as cross‐present these to Th1 CD4 and CD8 T cells.^63^, ^64^, ^65^ In addition to the genetic predisposition of study participants (e.g., HLA alleles and genetic polymorphisms^66^, ^67^, ^68^, ^69^, ^70^), the quality of DCs injected is an important factor that determines the outcomes of the clinical trials. This has been demonstrated by the close correlations between levels of IL‐12 produced by vaccine candidates and better disease outcomes during the clinical trials in cancer patients by our group^41^ and others.^71^

To date there have been at least 16 early‐phase clinical trials of DC‐based HIV vaccines.^51^, ^52^, ^53^, ^54^, ^55^, ^56^, ^57^, ^58^, ^59^, ^60^ DCs were uniformly well tolerated, but the immune response was insufficient to control the virus and achieve a “functional cure,” which indicates that although promising, there are factors, as of yet unknown, which affect the outcomes of these trials. Although there is a high variability in terms of the immunization routes, immunogens, participant profiles, and number of DCs injected, all protocols used monocytes as the source of DCs.^72^ In that respect, we and others have previously shown that HIV infection is associated with aberrant platelet activation^73^, ^74^, ^75^, ^76^, ^77^ and results in increased levels of circulating PMCs.^12^, ^13^, ^14^, ^15^, ^16^ Interaction of monocytes with activated platelets drove the monocytes toward a proinflammatory, promigratory phenotype that accentuated neuroinflammation.^14^ Based on these observations, we hypothesized that platelet‐induced functional defects of monocytes, dampen the immunogenicity of these cells even after their subsequent differentiation into DCs.

Indeed, we show that DCs which were complexed to platelets or were exposed to platelet‐derived biomolecules expressed significantly lower levels of various surface proteins important for DC function, such as, antigen uptake receptors (CD206, DC‐SIGN, CD613, CD16, CCR5), antigen presentation (HLADR), T cell costimulation (CD40, CD80, CD86), and chemokine receptor involved in migration lymph node (CCR7). These cells also showed a significant loss in CLEC9A expression, which is a marker of DCs proficient at antigen cross‐presentation. This functional characteristic is especially critical for induction of CTL immune responses against pathogens that do not productively infect DCs, including HIV.

Further two cytokine cocktails were used to mature DCs derived from PMCs, standard cocktail and type 1 polarizing cocktail (developed by our group and consisting of IL‐1β, TNFα, IFNγ, IFNα, and Poly I:C). Although the in vitro monocyte derived DCs (MoDCs) do not exactly correspond to any particular in vivo subset of DCs, MoDCs are considered more similar to conventional DCs in humans, rather than the other subsets of DCs such as plasmacytoid DCs.^78^, ^79^, ^80^, ^81^ In steady state, most of the circulating DCs are derived from bone marrow precursors and are independent of monocytes.^82^, ^83^, ^84^, ^85^ However, during infection or inflammation, monocytes mobilize to generate DCs.^1^, ^86^, ^87^, ^88^, ^89^ Rarity of this population in whole blood was the main challenge in investigating human DCs, but this obstacle was overcome when monocytes were found to differentiate into DCs via use of IL‐4 and GM‐CSF and this culture method has been used as a platform for much of our understanding of human DC biology and their role in immunity and tolerance.^90^, ^91^, ^92^ This was followed by development of the gold standard cytokine cocktail comprising IL‐1β, IL‐6, TNFα, and PGE_2_, as maturation signals for DCs;^93^, ^94^ however, MoDCs derived using this cocktail proved ineffective for cancer immunotherapy.^95^, ^96^, ^97^, ^98^ In this regard, we have previously shown that, PGE_2_, which is found to be elevated during inflammation, induced regulatory T cell (Treg) attracting properties in mature DCs via production of CCL22.^99^ PGE_2_ and its key synthesizing enzyme, COX2, had negative regulatory effect on type 1 effector cell priming during chronic inflammation.^100^ This effect of PGE_2_ was counteracted by IFNα. In addition, IFNγ, IL‐1β, and TNFα aided in overcoming maturation associated cellular exhaustion and resulted in yield of more stable, type 1 polarized DCs. These DCs also secreted higher levels of IL‐12 (an attribute absent in standard DCs), which is critical for induction of type 1 immunity. A recent study, which underscores the potential of type 1 DCs against HIV, showed that type 1 DCs drove antigen‐specific reversal of HIV latency.^51^ Overall, the type 1 cocktail, has been shown to be superior to the standard cocktail in that it can generate significantly higher levels of CTL immune responses.^40^ However, despite such a potent cytokine stimulation, in this study, type 1 DCs showed significantly reduced expression of three markers, CD80, CCR7, and CCR5 when derived from platelet complexed monocytes, indicating that platelets are associated with phenotypic dysregulation of monocytes differentiation into DCs. Interestingly, platelets enhanced the ability of DCs to migrate across endothelial barrier (despite reduced expression of CCR7).

In order to further delineate the role of platelets especially their activation status, more treatment groups were included in the subsequent experiments, such as MoDCs differentiated in presence of not activated platelets or platelets activated using thrombin. In addition, cells isolated from HIV infected individuals were also used to assess if these cells exhibit similar defects upon interaction with activated platelets. Experiments were performed to measure two crucial experimental readouts of DC function, cytokine secretion and naïve T cell stimulation. Results indicate that type 1 DCs derived from PMCs secreted reduced levels of IL‐12. In support of these results, Ki et al. showed that in an in vitro model, human platelet transfusion resulted in reduced expression of CD40, CD80, and CD86 in BDCA3^+^ DCs and also suppressed the production of IL‐12 by myeloid DCs.^22^, ^23^ Similar results were also described by Kissel et al. in MoDCs.^101^ Interestingly, DCs in individuals with immune thrombocytopenia exhibit increased expression of CD80, CD86, and also secrete higher levels of IL‐12.^102^ Further these cells also show reduced ability to stimulate regulatory T cells,^103^ indicating that platelets do regulate DC phenotype and polarization.

Finally, we measured the ability of PMC derived DCs to stimulated naïve CD4 and CD8 T cells against HIV antigens and found that MoDCs that had interacted with activated platelets were less efficient at stimulating both naïve CD4 and CD8 T cells, independent of HIV status. Cumulatively, these findings demonstrate that interaction with activated platelets in the form of PMCs, results in increased migration of immunogenically inferior DCs. Thus, it is possible that these cells might induce T cell anergy, instead of generating viable antigen‐specific immune responses against HIV.

During the generation of DCs for immunotherapy, specifically when monocytes are used as their source, contamination of the preparation with residual platelets has always been a concern^104^, ^105^; however, very few studies have investigated this phenomenon in depth. Sitia et al. showed that antiplatelet therapy prevents hepatocellular carcinoma and improves survival in a mouse model of chronic hepatitis B infection.^106^ Platelets were found to constrain T cell immunity through GARP‐TGFβ axis and platelet specific deletion of GARP‐encoding gene blunted TGFβ activity at tumor site and potentiated protective immunity against both melanoma and colon cancer.^107^ A recent study by Hilt et al. alludes that platelet‐derived TGFβ and β_2_M have opposing roles in monocyte differentiation toward a pro‐reparative VS proinflammatory phenotype with respect to tissue injury response.^108^ Our findings align very well with the findings from these reports albeit with respect to anti‐HIV immune responses and show long‐lasting repercussions of PMC formation on DC function. The next obvious step in understanding the polygonal platelet‐monocyte/DC interaction is to unravel the mechanisms underlying this phenomenon by blockade of platelet monocyte/DC complexes through the p‐selectin:PSGL1 and JAM‐C:Mac‐1 receptor ligand pairs,^14^, ^29^ as well as selective inhibition of platelet biomolecules such as CD40L, TGFβ, and β_2_M. Functional analysis of monocytes and DCs from individuals who are prescribed antiplatelet therapies such as Clopidogrel or Prasugrel (which disrupt platelet‐leukocyte interaction)^109^, ^110^ might also provide further novel insights about immune‐modulation by platelets. Altogether, we postulate that DCs derived from monocytes that are not complexed to platelets might significantly improve the immunogenic outcomes for DC‐based vaccine candidates against HIV as well as spontaneous control of the virus. Importantly, our findings also have significant implications for other chronic diseases such cancer, autoimmune disorders and merit further investigation.

## AUTHORSHIP

The authors contributed in the following manner: M.V.S.: conceptualization, investigation, data curation, writing the original draft, and revision; S.S.: data curation, manuscript revision, and editing; S.R.S.: data curation, manuscript revision, and editing; E.A.W.: data curation, manuscript revision, and editing; V.B.S.: data curation, manuscript revision, and editing; P.K.: conceptualization, manuscript revision, and editing; and S.B.M.: conceptualization, manuscript revision, and editing.

## DISCLOSURES

The authors declare no conflicts of interest.

## Supporting information

Supporting Information.

Supporting Information.

Supporting Information.
