# Supplementary figures and images for: Monocytes complexed to platelets differentiate into functionally deficient dendritic cells
Source: J Leukoc Biol. 2020 Jul 14;109(4):807–20. doi: 10.1002/JLB.3A0620-460RR (PMC7854860; doi:10.1002/JLB.3A0620-460RR)

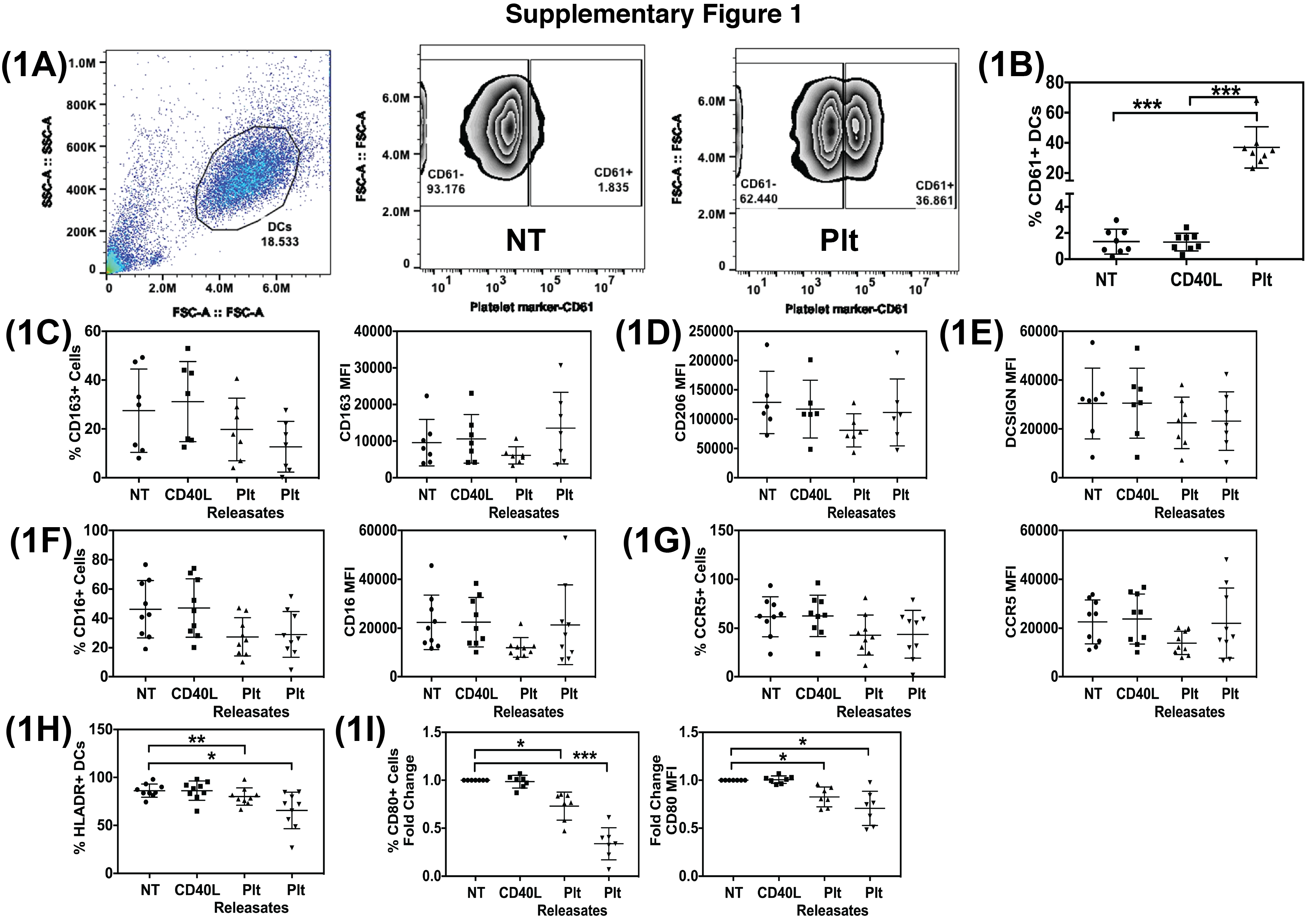

Supplement: Supplementary file 1 — Supporting Information. [file JLB-109-807-s001.tif]

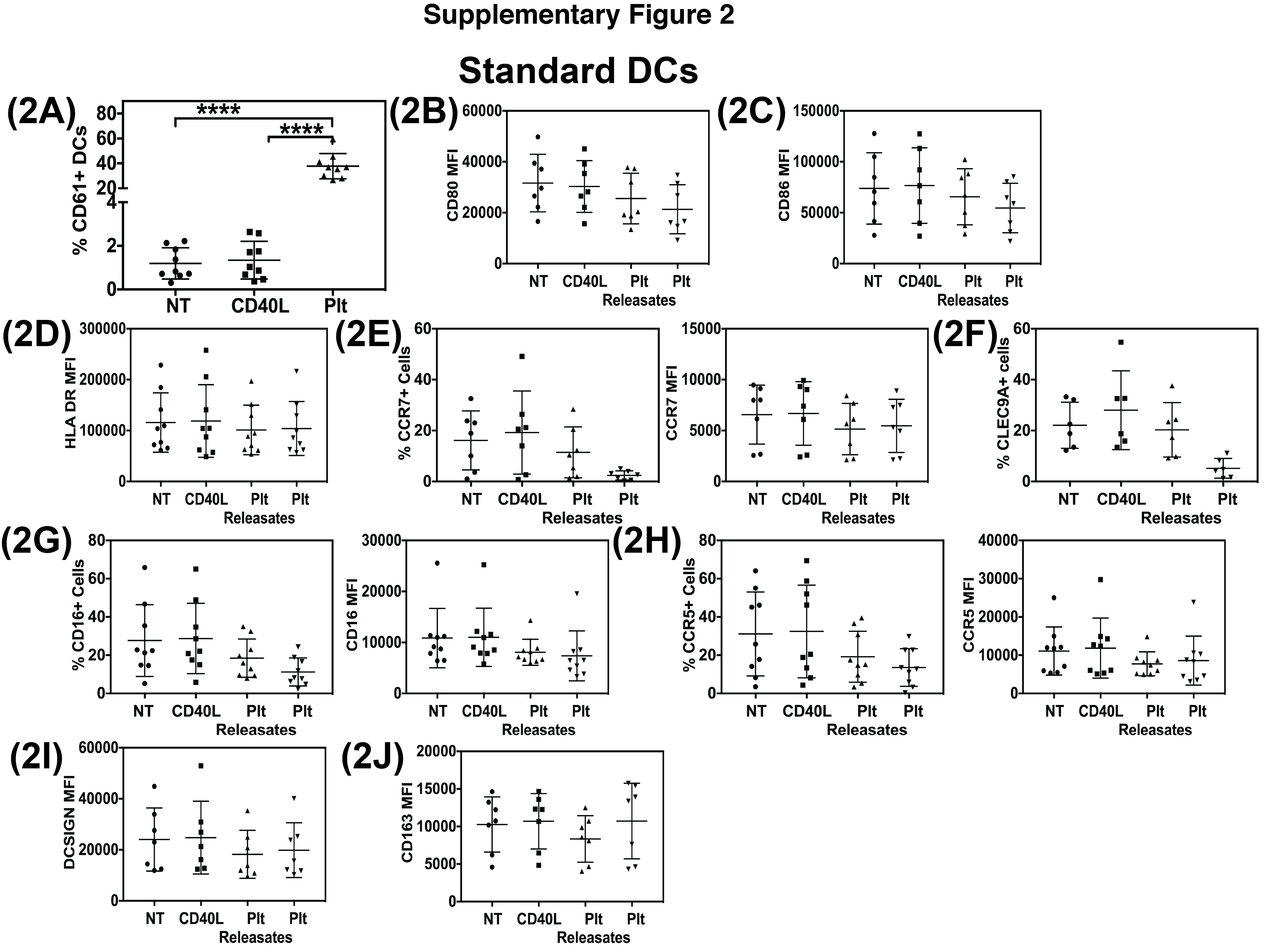

Supplement: Supplementary file 2 — Supporting Information. [file JLB-109-807-s002.tif]

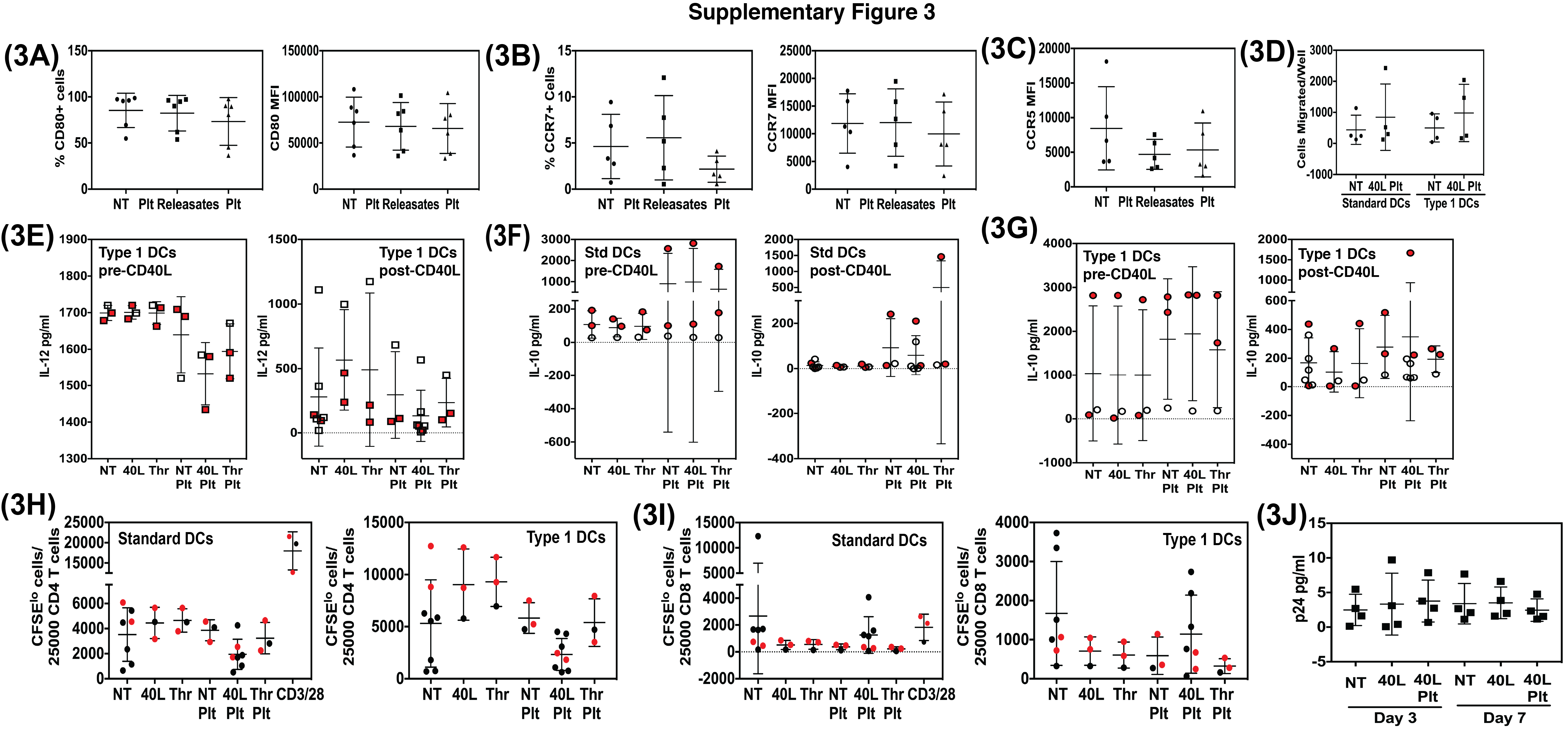

Supplement: Supplementary file 3 — Supporting Information. [file JLB-109-807-s003.tif]
